# Fabrication of pH-Responsive Zn^2+^-Releasing Glass Particles for Smart Antibacterial Restoratives

**DOI:** 10.3390/molecules27217202

**Published:** 2022-10-24

**Authors:** Fan Deng, Hirohiko Sakai, Haruaki Kitagawa, Tomoki Kohno, Pasiree Thongthai, Yuhan Liu, Ranna Kitagawa, Gabriela L. Abe, Jun-ichi Sasaki, Satoshi Imazato

**Affiliations:** 1 Department of Biomaterials Science, Osaka University Graduate School of Dentistry, Yamadaoka, Suita 565-0871, Japan; 2 Department of Advanced Functional Materials Science, Osaka University Graduate School of Dentistry, Yamadaoka, Suita 565-0871, Japan; 3 Department of Operative Dentistry, Faculty of Dentistry, Chulalongkorn University, Pathum Wan, Bangkok 10330, Thailand; 4 Department of Stomatology, Aviation General Hospital of China Medical University and Beijing Institute of Translational Medicine, Chinese Academy of Sciences, Beijing 100012, China; 5 Department of Restorative Dentistry and Endodontology, Osaka University Graduate School of Dentistry, Yamadaoka, Suita 565-0871, Japan

**Keywords:** dental materials, pH-responsive ion-releasing glass, smart antibacterial restorative, zinc, root surface caries

## Abstract

The on-demand release of antibacterial components due to pH variations caused by acidogenic/cariogenic bacteria is a possible design for smart antibacterial restorative materials. This study aimed to fabricate pH-responsive Zn^2+^-releasing glass particles and evaluate their solubilities, ion-releasing characteristics, and antibacterial properties in vitro. Three kinds of silicate-based glass particles containing different molar ratios of Zn (PG-1: 25.3; PG-2: 34.6; PG-3: 42.7 mol%) were fabricated. Each particle was immersed in a pH-adjusted medium, and the solubility and concentration of the released ions were determined. To evaluate the antibacterial effect, *Streptococcus mutans* was cultured in the pH-adjusted medium in the presence of each particle, and the bacterial number was counted. The solubility and concentration of Zn^2+^ released in the medium increased with a decrease in medium pH. PG-3 with a greater content of Zn demonstrated higher concentrations of released Zn^2+^ compared with PG-1 and PG-2. PG-2 exhibited bactericidal effects at pH 5.1, whereas PG-3 demonstrated bactericidal effects at pH values of 5.1 and 6.1, indicating that PG-3 was effective at inhibiting *S. mutans* even under slightly acidic conditions. The glass particle with 42.7 mol% Zn may be useful for developing smart antibacterial restoratives that contribute to the prevention of diseases such as caries on root surfaces with lower acid resistance.

## 1. Introduction

Several studies have been conducted to develop antibacterial restorative materials. One of the effective approaches is to release the antibacterial components using a carrier, such as silver nanoparticles [1,2], ion-releasing glass fillers [3,4,5], and antimicrobial-loaded polymer particles [6,7,8]. However, these approaches typically employ a simple design to exhibit the release of antimicrobials under non-controlled conditions [9]. Moreover, the ecological perturbations via the antimicrobial effects displayed by the continuous delivery of these agents exceed thresholds, disrupting homeostasis in the oral environment. Oral microorganisms form a complex ecosystem that thrives in the dynamic environment in a symbiotic relationship with the human host [10]. Several kinds of bacteria—called early (initial) colonizers—associated with oral health have substantial ecological advantages. These organisms bind more avidly to salivary-pellicle-coated teeth, demonstrate more rapid growth, antagonize pathogens via multiple mechanisms, and help maintain microbial homeostasis and stability [10,11]. Nevertheless, once dental plaque is formed on the surfaces of the teeth or materials, the pH value of the plaque decreases owing to the acids produced by acidogenic bacteria, leading to tooth demineralization and dental caries. Therefore, providing restorative materials with an on-demand release ability to effectively supply antimicrobial components is beneficial when these acidogenic bacteria produce acids in the plaque.

Several pH-responsive ion-releasing technologies have been reported so far [12,13,14]. Zinc is one of these ions known to inhibit oral bacteria. Liu et al. reported that the release of Zn^2+^ from the BioUnion filler, a glass powder composed of silicon dioxide (SiO_2_), zinc oxide (ZnO), calcium oxide (CaO), and fluorine (F), accelerated under acidic conditions at a pH of 4.5 [15,16,17]. Thus, such technology enables the on-demand release of antimicrobial components from restorative materials. Liu et al. also reported that the acidity-induced release of Zn^2+^ from the glass ionomer cement (GIC) containing BioUnion filler (Caredyne^®^ Restore, GC Corp., Tokyo, Japan) effectively inhibited the growth and adherence of acidogenic bacteria [17].

A pH in the range of 6.7–7.3 is typically maintained in the oral cavity via the perfusion of saliva with pH-buffering capabilities. Nevertheless, the pH of the dental plaque formed on tooth surfaces decreases with an intake of carbohydrates in a critical pH range of 5.2–5.5, resulting in enamel demineralization [18]. Particularly in the elderly, dental caries frequently occur on the root surface owing to gingival recession and root surface exposure. As the exposed cementum and dentin of the root have lower acid resistance than the enamel, the critical pH of the root surface has been reported to be higher than 6 [19,20,21,22]. The BioUnion filler and GIC containing BioUnion filler in the acid at pH 4.5 were found to release sufficient amounts of Zn^2+^ and effectively inhibit the growth and adherence of oral bacteria. However, a novel technique that effectively releases Zn^2+^ even with a minimal decrease in pH is more desirable for applications to the root surface with lower acid resistance. In this study, we fabricated novel pH-responsive glass particles with varying Zn contents. The purpose of this study was to evaluate their solubility, ion-releasing characteristics, and antibacterial properties in vitro.

## 2. Results

### 2.1. Characterization of Glass Particles

**Figure 1:**
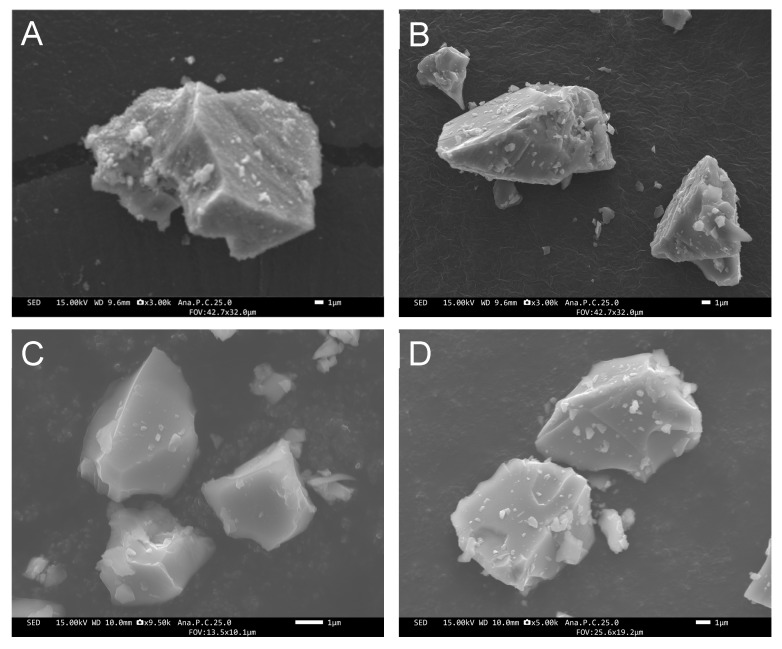
FE-SEM images of the glass particle surfaces of (**A**) Cont, (**B**) PG-1, (**C**) PG-2, and (**D**) PG-3.

**Figure 2:**
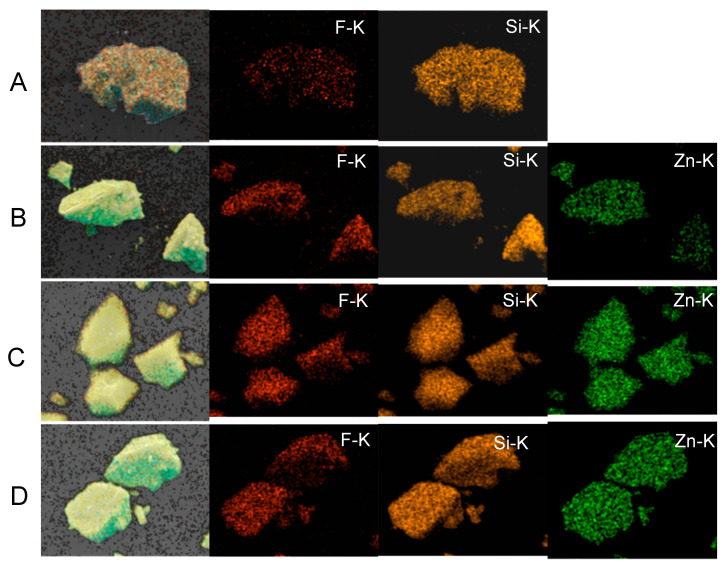
Elemental mapping images (EDS) of the four glass particles. (**A**) Cont, (**B**) PG-1, (**C**) PG-2, and (**D**) PG-3.

**Figure 3:**
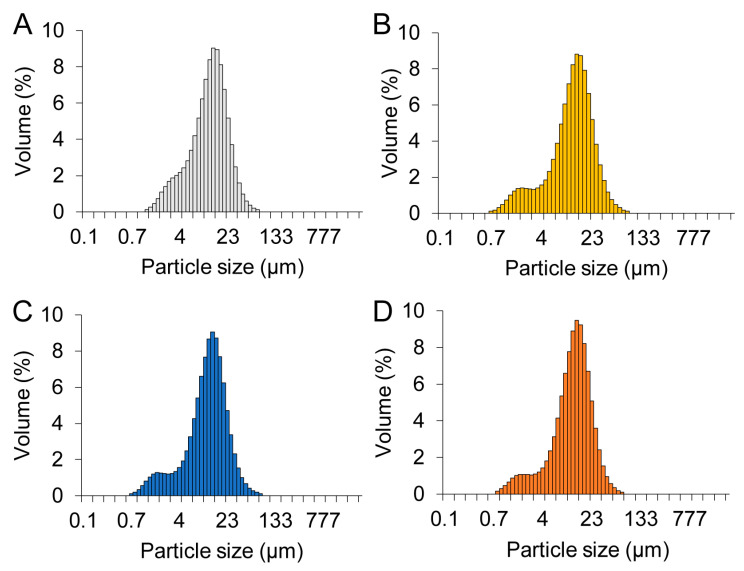
Particle size distribution (PSD) of the four glass particles. (**A**) Cont, (**B**) PG-1, (**C**) PG-2, and (**D**) PG-3.

All of the prepared glass particles were of irregular shapes (Figure 1). The median diameter of Cont, PG-1, PG-2, and PG-3 was 11.2, 11.3, 10.9, and 11.3 µm, respectively. As shown in Figure 2, the elemental mapping images revealed that the elements were evenly dispersed in the four glass particles. Their elemental composition is shown in Table 1. According to XRF analysis, Si, Zn, and F were detected in Cont with a mol fraction of 70.8, 0, and 15.1%, respectively; in PG-1 with 42.9, 25.3, and 17.0%, respectively; in PG-2 with 33.4, 34.6, and 16.0%, respectively; and in PG-3 with 29.2, 42.7, and 10.5%, respectively. No remarkable difference in composition was observed between the preparation and result of the XRF analysis. The particle size distribution of each glass particle is shown in Figure 3. No significant difference was observed in the particle size range and distribution among the four groups.

### 2.2. Solubility and Ion Release Property of Glass Particles in pH-Adjusted Media

**Figure 4:**
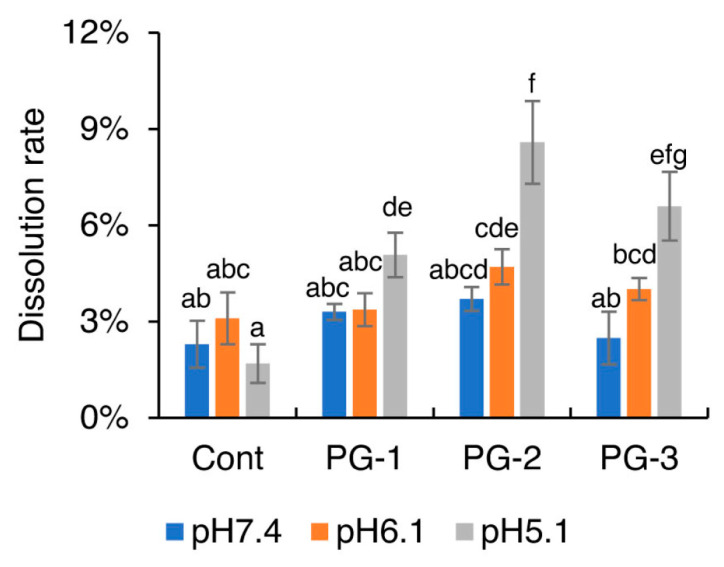
Solubilities of the four glass particles: Cont., PG-1, PG-2, and PG-3 in pH-adjusted BHI broth. Bars represent the standard deviation of the three replicates. a–g: different letters indicate significant differences (*p* < 0.05, ANOVA, Tukey’s HSD test).

**Figure 5:**
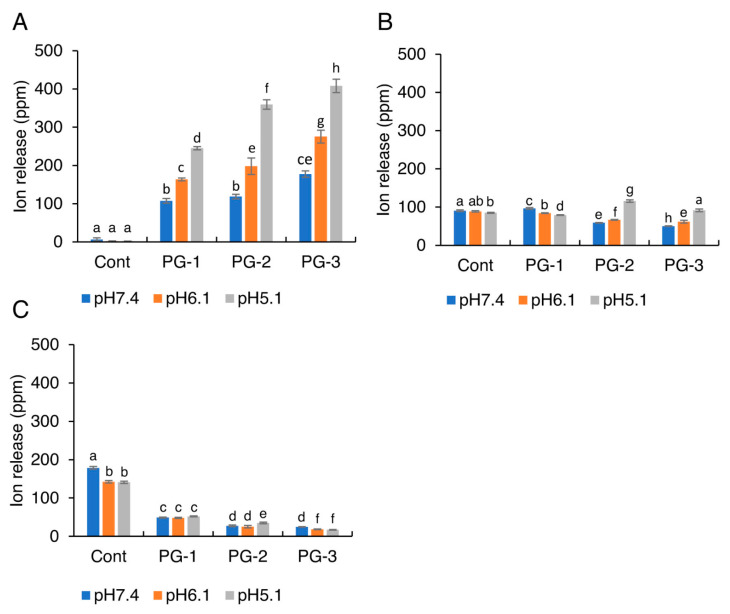
The concentrations of (**A**) Zn^2+^, (**B**) SiO_3_^2−^, and (**C**) F^−^ released from the glass particles into the pH-adjusted BHI broth. Bars represent the standard deviations of the six replicates. a–h: letters indicate significant differences (*p* < 0.05, ANOVA, Tukey’s HSD test).

Figure 4 shows the solubility of each glass particle in pH-adjusted BHI broth (pH 7.4, 6.1, or 5.1). When the pH value was at 6.1 or 7.4, no significant difference among the glass particles was observed. When the pH decreased to 5.1, the solubility of the glass particles containing Zn showed a significant increase. Furthermore, no significant difference in the solubility was observed between PG-2 and PG-3 under each pH condition. The profiles of Zn^2+^, SiO_3_^2−^, and F^−^ released from glass particles into the pH-adjusted BHI broth are shown in Figure 5. With a decrease in pH values, the release of Zn^2+^ from Cont., PG-1, PG-2, and PG-3 increased significantly. With an increase in Zn content in Cont., PG-1, PG-2, and PG-3, the release of Zn^2+^ increased at each pH value, whereas the release of F^−^ significantly decreased.

### 2.3. MICs and MBCs of Zn^2+^, SiO_3_^2−^, and F^−^ for S. mutans

The MICs and MBCs of Zn^2+^, SiO_3_^2−^, and F^−^ against *S. mutans* are shown in Table 2. For Zn^2+^, the MIC and MBC for *S. mutans* were 125 and 250 ppm, respectively. For SiO_3_^2−^, the MIC and MBC for *S. mutans* were greater than 500 ppm. Additionally, for F^−^, the MIC for *S. mutans* was 125 ppm, whereas the MBC was greater than 500 ppm.

### 2.4. Antibacterial Activity of Glass Particles against S. mutans

**Figure 6:**
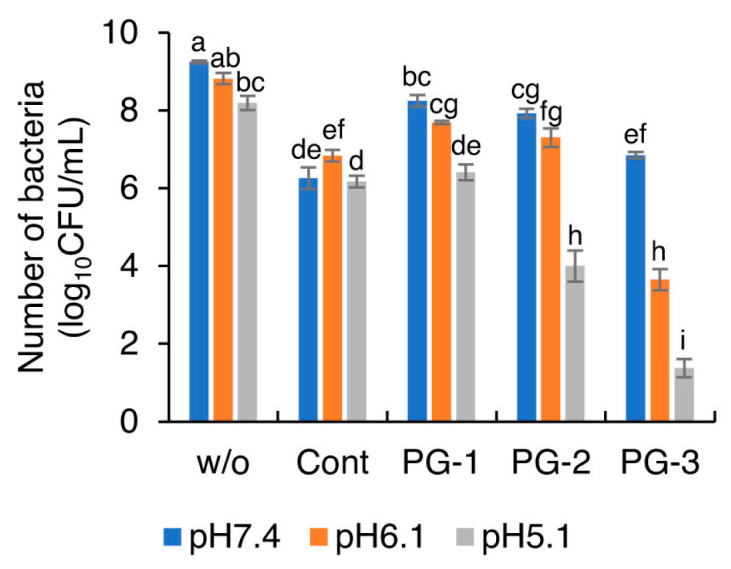
Number of viable *S. mutans* after 24 h incubation at pH 5.1, 6.1, and 7.4 with four glass particles. Bars represent the standard deviations of six replicates. a–i: letters indicate significant differences (*p* < 0.05, ANOVA, Tukey’s HSD test). w/o, without any powders.

Figure 6 shows the number of viable bacteria after incubation after immersing Cont, PG-1, PG-2, and PG-3 in the pH-adjusted BHI broth. After 24 h of anaerobic incubation, the colony counts of viable *S. mutans* in the presence of glass particles were significantly lesser than tho without any particles at each pH value. No significant difference was found in the number of surviving cells in the presence of Cont (approximately 6.2 log_10_CFU/mL) between pH 7.4 and 6.1. With a decrease in the pH value, the viable number of *S. mutans* significantly decreased in the presence of PG-1, PG-2, and PG-3. The number of viable cells in the presence of PG-1 (pH 5.1), PG-2 (pH 5.1), and PG-3 (pH 6.1 and pH 5.1) was significantly smaller than the initial number of bacteria (approximately 7.0 log_10_CFU/mL), indicating that they exhibited bactericidal effects at the corresponding pH values.

## 3. Discussion

Zn^2+^ has multiple inhibitory effects on intact bacterial cell activities such as glycolysis, glucosyltransferase production and polysaccharide synthesis, transmembrane proton translocation, and acid tolerance [23]. It can enhance the proton permeabilities of bacterial cell membranes, reduce acid production, and inhibit cell metabolism [17,24]. Fluoride is assumed to be anticariogenic via various mechanisms including the reduction in demineralization, enhancement in remineralization, interference of pellicle and plaque formation, and inhibition of microbial growth and metabolism [25,26]. As described above, the BioUnion filler is composed of SiO_2_, ZnO, CaO, and F. Ca^2+^ and F^−^ enhance remineralization and inhibit demineralization [27]. However, the inhibitory effect of Ca^2+^ on the growth of oral bacteria is not as strong as that of Zn^2+^ and F^−^ [17]. Therefore, in this paper, three types of experimental glasses containing Zn and F (i.e., without the addition of Ca) were studied to strengthen their antibacterial activity compared with the BioUnion filler.

As intermediates contributing to field strength, Zn^2+^ behaves either as a modifier or a network-former in glass [28]. As a modifier, Zn^2+^ exists as charged single ions in the cross-linked glass network. It disrupts the regular bonding between the glass-forming components and oxygen through non-bridging oxygen (NBO; Si-O^−^M^+^, where M^+^ is a modifier ion) [29]. This decreases the relative quantity of strong bonds in glass. As a network-former, Zn^2+^ enters the silicate network by forming Si-O-Zn bonds. The mechanism of glass dissolution involves the ion exchange (protons for modifier ions) and acid hydrolysis of Si-O-Zn bonds. Zinc-releasing silicate glasses are stable at physiological pH, but show accelerated dissolution under acidic conditions [30]. This suggests that zinc enters the silicate network to a much greater extent, with a majority or potentially all zinc forming Si-O-Zn bonds [29]. By adding zinc oxide to silicate glasses, modifying the ion-releasing behavior in response to changes in the pH environment is possible. Consequently, the glasses are more stable at neutral pH levels, but quickly dissolve in acidic conditions. In this study, the solubility of glass particles in acids increased with an increase in zinc content. According to the ICP results in our study, the release of Zn^2+^ increased with an increase in the Zn content of glass. Additionally, all glasses containing Zn released a greater amount of Zn^2+^ with a decrease in pH. These results can be attributed to the degradation mechanisms discussed above. A higher amount of zinc content was related to higher NBO and Si-O-Zn bonds in the glass structure, thus releasing a larger amount of Zn^2+^ in aqueous solution, particularly in acidic conditions. The control glass showed the greatest amount of fluoride release among the four groups despite an equal fluoride content. With the increase in Zn in the glass (PG-1 < PG-2 < PG-3), the release of F^−^ from the glass decreased (PG-1 > PG-2 > PG-3). This may be explained by the complexing reaction: Zn^2+^ + HF = ZnF^+^ + H^+^, which occurs in the aqueous solution [31]. Most of the fluoride released from glass ionomer cements is known to be in the complexed form rather than as “free” F^−^ [32]. Thus, it can be assumed that the existence of Zn in glass particles interferes with the release of F.

To determine the inhibitory effect of the components in the glass particles, the MICs and MBCs of Zn^2+^, SiO_3_^2−^, and F^−^ against *S. mutans* were measured via a microdilution assay. An MBC of 4.8 mg/mL (480 ppm) for NaF against *S. mutans* has been reported [33]. Our previous study reported an MIC of 128 ppm and MBC of 2048 ppm for F^−^ against *S. mutans* [17]. The MIC of NaF against *S. mutans* in this study was 125 ppm, while the MBC was over 500 ppm. All of the concentrations of fluoride released from the four groups were much lower than the MBC of F^−^. The concentrations of fluoride released from control glass at the three pH levels were higher than the MIC of F^−^, while the concentrations of fluoride released from PG-1, PG-2, and PG-3 were lower than the MIC of F^−^. Therefore, control glass exhibited inhibitory effects against bacterial growth (the number of bacteria was the same/similar to the initial concentration of bacteria) as fluoride has a direct inhibitory effect on the metabolic activity of cariogenic bacteria. Hernández-Sierra et al. reported an MIC range of 500 ± 306.18 µg/mL (500 ± 306.18 ppm) and MBC of 500 μg/mL (500 ppm) for ZnO-NPs against *S. mutans* [34]. Yu et al. reported an MIC of 0.156 mg/mL (156 ppm) and MBC of 0.312 mg/mL (312 ppm) for ZnO-NPs against *S. mutans* [35]. Salts (zinc chloride and zinc gluconate) and zinc oxide nanoparticles (ZnO-NPs) are the most common forms used in the evaluation of MICs and MBCs [36], while zinc nitrate (Zn(NO_3_)_2_) was rarely reported. In this study, the MIC of Zn(NO_3_)_2_ for *S. mutans* was 125 ppm, while the MBC was 250 ppm. The concentrations of Zn^2+^ released at pH 5.1 and 6.1 for PG-1, PG-2, and PG-3 were greater than the MIC (125 ppm) against *S. mutans*, whereas the concentrations of Zn^2+^ released at pH 7.4 of PG-1 and PG-2 were lower than this value. The concentrations of Zn^2+^ released from PG-1 (pH 5.1), PG-2 (pH 5.1), and PG-3 (pH 5.1, 6.1) were higher than the MBC (250 ppm) against *S. mutans*, whereas the concentrations of Zn^2+^ released from PG-1 (pH 6.1 and 7.4), PG-2 (pH 6.1 and 7.4), and PG-3 (pH 7.4) were lower than this value. Liu et al. reported that the concentration of Zn^2+^ released from the BioUnion filler in acetic acid at pH 5.5 was 166.9 ± 11.2 ppm [15]. In this study, the concentration of Zn^2+^ released from PG-3 in the medium adjusted to pH 6.1 was 275.4 ± 17.0 ppm, higher than that of Zn^2+^ released from BioUnion filler at pH 5.5. As supported by these results, PG-3 demonstrated bactericidal effects (the number of bacteria was lower than the initial concentration of bacteria) under pH of 5.1, as well as 6.1, owing to the effective release of Zn^2+^ even under slightly acidic conditions. This result suggests that the release of Zn^2+^ from PG-3 would prevent caries on root surfaces with low acid resistance.

When the restorative materials incorporating the glass particles are exposed to acids, the increased dissolution might adversely affect the physical properties of the materials. An example is GICs wherein glass particles can be incorporated. The chemical composition of the GIC powder has been substantially modified to improve the handling characteristics and mechanical properties. These modifications are based on the hypothesis that a higher number of poly-salt bridges can be formed in the GIC matrix owing to the increased chemical affinity between the filler particles and GIC matrix. This in turn enhances the mechanical properties of the material, making it suitable for posterior dental restoration. In addition, the solubility of GICs is closely related to the ion-releasing behavior, and thus has considerable influence on the antibacterial action. Hence, the particle size and composition of the glass particles influence the antibacterial and physical properties of GICs [37]. The median diameter of glass particles in this study was approximately 10 μm. A previous study reported that the compressive strengths of the GICs incorporating Zn^2+^-released glass particles with a median diameter of 4.5–5.5 µm did not decrease even after 28-day aging [15]. Some studies indicated that GICs continued the maturation of the acid–base reaction after mixing over time [38] and, consequently, the compressive strength slightly increased. Therefore, we cannot assume that the adverse effects on physical properties are negligible. Furthermore, in this study, the ion release and antibacterial effect against *S. mutans* were evaluated only for the first 24 h. Further investigations are required to evaluate ion release over longer periods and assess the antibacterial/anti-plaque effects against multi-species bacteria under simulated oral environments.

## 4. Materials and Methods

### 4.1. Fabrication of Glass Particles

Three types of silicate-based glasses containing zinc were fabricated and named PG-1, PG-2, and PG-3. A glass particle without zinc was used as the control group (Cont.). The glass particles were prepared by melting SiO_2_, ZnO, F, and other raw materials in a platinum crucible for 1 h at 1300–1500 °C followed by immediate quenching in distilled water. A sieve was used for classification and particles with a median diameter of approximately 10 µm were obtained. The prospective compositions of Cont., PG-1, PG-2, and PG-3 during preparation are listed in Table 3. All of the glass particles were sterilized with ethylene oxide gas.

### 4.2. Characterization of Glass Particles

The morphology and elemental compositions of each glass particle were observed and analyzed via field emission scanning electron microscopy and energy-dispersive spectroscopy (FE-SEM/EDS; JSM-F100, JEOL Ltd., Tokyo, Japan). The chemical compositions were also analyzed via X-ray fluorescence (XRF) analysis. Further, the particle size distribution (PSD) of each glass particle was observed via a particle size analyzer (Partica LA-960V2, HORIBA, Kyoto, Japan).

### 4.3. Solubility and Ion Release Evaluation of Glass Particles in pH-Adjusted Media

The solubility and ion release property of each glass particle in the pH-adjusted media were evaluated. Here, 50 mg of each particle was immersed in 10 mL of brain heart infusion (BHI) broth (Becton Dickinson, Sparks, MD, USA), whose pH was adjusted by hydrochloride acid (Kanto Chemical Co., Inc, Tokyo, Japan) at 7.4, 6.1, and 5.1. After storage at 37 °C for 24 h and gyratory shaking at 100 rpm, the particles were filtered through a 0.22 µm syringe filter (Merck Millipore Ltd., Carrigtwohill, Ireland), which was weighed beforehand (W_1_ mg) and dried at 110 °C for 48 h. The total weight (W_2_ mg) of the undissolved particles and the syringe filter was then measured, and the solubility of each particle was calculated according to the following equation:

Solubility (%) = (50 + W_1_ − W_2_)/50 × 100

To determine the profiles of Zn^2+^, SiO_3_^2−^, and F^−^ released from the glass particles into pH-adjusted BHI broth, 20 mg of each glass particle was placed in one well of a 96-well microplate. The particles in the wells were immersed in 200 μL of pH-adjusted BHI broth. After storage at 37 °C for 24 h and gyratory shaking at 100 rpm, the suspensions were diluted with 9.8 mL distilled water, after which the concentrations of Zn^2+^ and SiO_3_^2−^ were measured using an inductively coupled plasma-optical emission spectrometer (ICP-OES; iCAP7200 ICP-OES Duo, Thermo Fisher Scientific, Cambridge, UK) and the concentration of F^−^ was determined using a fluoride ion electrode (FIE; 6561S-10C, HORIBA, Kyoto, Japan). The experiments were repeated six times.

### 4.4. Measurement of MICs and MBCs of Zn^2+^, SiO_3_^2−^, and F^−^ for Streptococcus mutans NCTC10449

*Streptococcus mutans* NCTC10449 from a stock culture was incubated in BHI broth and on BHI agar plates (Becton Dickinson) at 37 °C for 24 h under anaerobic conditions. To evaluate the concentrations of Zn^2+^, SiO_3_^2−^, and F^−^ that could effectively inhibit the growth of *S. mutans*, the minimum inhibitory concentrations (MICs) and minimum bactericidal concentrations (MBCs) of each ion against *S. mutans* were measured with a microdilution assay. Briefly, 1 mg/mL of Zn(NO_3_)_2_, Na_2_SiO_3_, and NaF (FUJIFILM Wako Pure Chemical Corporation, Osaka, Japan) were used as standard solutions for Zn^2+^, SiO_3_^2−^, and F^−^, respectively. Here, 50 μL of standard solutions with a concentration of 0.49–1000 ppm obtained after serial twofold dilutions was dropped into the wells of a 96-well microplate. Next, 50 μL of *S. mutans* suspension, adjusted to 2.0 × 10^7^ colony-forming units (CFU)/mL with 2× BHI broth, was added into each well with the standard solutions. Then, the microplates were anaerobically incubated at 37 °C for 24 h. The turbidity of the suspensions was observed visually and the MIC, which is the lowest concentration that prevents visible bacterial growth, was determined. Then, 20 μL of the clear samples was inoculated on agar plates. After anaerobic sub-culturing for 24 h at 37 °C, the MBC, which is the lowest concentration of the antimicrobial agent that kills the bacterium, was determined using the agar plates with no bacterial colonies. These experiments were repeated five times.

### 4.5. Evaluation of Antibacterial Activity of Glass Particles

The *S. mutans* NCTC10449 suspension was adjusted to approximately 1.0 × 10^8^ CFU/mL in BHI broth. A total of 20 mg of each glass particle was placed in a well of a 96-well microplate. Then, 180 μL of pH-adjusted BHI broth (pH 7.4, 6.1, and 5.1) and 20 μL of *S. mutans* suspension (approximately 1.0 × 10^8^ CFU/mL) were added to it. After anaerobic incubation at 37 °C for 24 h with gyratory shaking at 100 rpm, 100 μL of the suspension was collected and diluted with 9.9 mL of BHI broth. The suspension was then serially diluted with BHI broth and inoculated on BHI agar plates. The plates were incubated anaerobically at 37 °C for 24 h, after which the number of formed colonies was determined. This experiment was repeated six times.

### 4.6. Statistical Analysis

Statistical analyses were performed using SPSS Statistics 25 (IBM, Chicago, IL, USA). The homogeneity of variances was confirmed initially. The results of solubility, ion release, and bacterial growth were statistically analyzed using analysis of variance (ANOVA) and Tukey’s honestly significant difference (HSD) test. *p* < 0.05 was considered to indicate significance in this study.

## 5. Conclusions

Glass particles with a pH-responsive Zn^2+^-releasing property were successfully fabricated to inhibit *S. mutans* growth under acidic conditions. The glass particles with 25.3 mol% Zn exhibited bactericidal effects at pH 5.1, whereas glass particles with 42.7 mol% Zn demonstrated killing effects at pH 5.1 and 6.1 due to the effective release of Zn^2+^ even under slightly acidic conditions. The glass particle with 42.7 mol% Zn may also be useful for developing smart antibacterial restoratives that contribute to the prevention of diseases such as root surface caries.

## Figures and Tables

**Table 1:** Elemental compositions of Cont, PG-1, PG-2, and PG-3 glasses (mol%) via XRF analysis.

Glass Composition	Cont.	PG-1	PG-2	PG-3
Si	70.8	42.9	33.4	29.2
Zn	0	25.3	34.6	42.7
F	15.1	17	16	10.5
Others	14.2	14.8	16	17.6

**Table 2:** Minimum inhibitory concentrations (MICs) and minimal bactericidal concentrations (MBCs) of Zn^2+^, F^−^, and SiO_3_^2−^ on *S. mutans* (in ppm).

	Zn^2+^	SiO_3_^2−^	F^−^
MIC	125	>500	125
MBC	250	>500	>500

**Table 3:** Prospective compositions of Cont, PG-1, PG-2, and PG-3 glasses (mol%) during preparation.

Glass Content	Cont.	PG-1	PG-2	PG-3
SiO_2_	68.7	45.7	35.5	30.5
ZnO	0	23	33.2	38.2
F	18.7	18.7	18.7	18.7
Others	12.6	12.6	12.6	12.6

## Data Availability

Not applicable.

## Sample Availability

Not available.
